# The 2003 Australian Breast Health Survey: survey design and preliminary results

**DOI:** 10.1186/1471-2458-8-13

**Published:** 2008-01-14

**Authors:** Elmer V Villanueva, Sandra Jones, Caroline Nehill, Simone Favelle, David Steel, Donald Iverson, Helen Zorbas

**Affiliations:** 1 National Breast Cancer Centre, Camperdown, NSW, Australia; 2 Gippsland Medical School, Monash University, Churchill, VIC, Australia; 3 Centre for Health Behaviour and Communication Research, University of Wollongong, Wollongong, NSW, Australia; 4 Faculty of Health and Behavioural Sciences, University of Wollongong, Wollongong, NSW, Australia; 5 School of Mathematics and Applied Statistics, University of Wollongong, Wollongong, NSW, Australia

## Abstract

**Background:**

The Breast Health Surveys, conducted by the National Breast Cancer Centre (NBCC) in 1996 and 2003, are designed to gain insight into the knowledge, attitudes and behaviours of a nationally representative sample of Australian women on issues relevant to breast cancer. In this article, we focus on major aspects of the design and present results on respondents' knowledge about mammographic screening.

**Methods:**

The 2003 BHS surveyed English-speaking Australian women aged 30–69 without a history of breast cancer using computer-assisted telephone interviewing. Questions covered the following themes: knowledge and perceptions about incidence, mortality and risk; knowledge and behaviour regarding early detection, symptoms and diagnosis; mammographic screening; treatment; and accessibility and availability of information and services. Respondents were selected using a complex sample design involving stratification. Sample weights against Australian population benchmarks were used in all statistical analyses. Means and proportions for the entire population and by age group and area of residence were calculated. Statistical tests were conducted using a level of significance of 0.01.

**Results:**

Of the 3,144 respondents who consented to being interviewed, 138 (4.4%) had a previous diagnosis of breast cancer and were excluded leaving 3,006 completed interviews eligible for analysis. A majority of respondents (61.1%) reported ever having had a mammogram and 29.1% identified mammography as being the best way of finding breast cancer. A majority of women (85.9%) had heard of the BreastScreen Australia (BSA) program, the national mammographic screening program providing free biennial screening mammograms, with 94.5% believing that BSA attendance was available regardless of the presence or absence of symptoms. There have been substantial gains in women's knowledge about mammographic screening over the seven years between the two surveys.

**Conclusion:**

The NBCC Breast Health Surveys provide a valuable picture of the knowledge of Australian women about a range of issues. The present analysis shows significant gains in knowledge and behaviours relating to mammographic screening, while identifying additional areas for targeted improvement, as in the need to better communicate with women about screening and diagnostic services. Further analysis of additional core topic areas (eg., incidence, mortality, risk and treatment) will provide equally noteworthy insight.

## Background

Breast cancer is the most common registerable cancer and is the leading cause of cancer-related death in Australian women [1]. In 2002, more than 12,000 were diagnosed with the disease and almost 2,600 died from it. By 2011, it is predicted that the number of new cases will exceed 14,800 [1].

In 1996, the National Breast Cancer Centre (NBCC) conducted the first national Breast Health Survey (BHS) to gain insight into the knowledge, attitudes and behaviour of a nationally representative sample of Australian women on issues relevant to breast cancer [2,3]. Key areas of investigation included respondents' understanding of the incidence of breast cancer, risk factors, early diagnosis, treatment and the availability and accessibility of information and services.

The results of the 1996 BHS were extremely useful in establishing baseline rates against which to assess the effectiveness of community information programs conducted by the NBCC and others. In addition, the findings suggested the areas upon which such programs might focus. For example, although it was clear that women were aware of the importance of breast cancer as a significant disease in the community, there was a need to emphasise that a diagnosis of breast cancer did not equate to certain and immediate death and that many women with breast cancer could be treated effectively [3].

In 2003, the NBCC undertook a second survey of a nationally representative sample of Australian women. The 2003 BHS retained a number of methods and questions from the 1996 BHS, as well as including questions on previously unexplored areas. In the following paper, we describe major aspects of the approach and design of the 2003 BHS and present results focusing on respondents' knowledge about mammographic screening.

## Methods

### Subjects and sampling design

The 2003 BHS surveyed English-speaking women aged 30–69 and residing in Australia during the last quarter of 2003 using computer-assisted telephone interviewing. Women were excluded if they had past history of breast cancer.

A random sample of telephone numbers was selected from the latest available electronic White Pages (Australia on Disk) [4]. Upon contacting a member of the selected household, women aged 30–69 with the most recent birthday [5] were invited to participate. Two stratifications were used: the first by State/Territory and the second to separate the capital cities from the rest of the State [6]. Stratum-specific sample sizes were set to allow for both the production of estimates for states and type of area and for state-by-state comparisons. The final sample size was 3,006 (Table 1). Sample weights were calculated to account for different selection probabilities and to produce estimates for the population of women ages between 30 and 69.

**Table 1:** Responding sample sizes by strata.

State/Territory	Capital City Sample	Rest of State Sample	Total Sample
New South Wales	320	200	520
Victoria	320	200	520
Queensland	250	199	449
South Australia	280	150	430
Western Australia	279	150	429
Tasmania	108	150	258
Northern Territory	120	80	200
Australian Capital Territory	200	-	200
Total	1,877	1,129	3,006

Weighting classes [7] were formed using five-year age groups for each of the 15 geographic strata. A check was made to ensure that each of the resulting cells had sufficient respondents to form stable weights. Weights were calculated using two sets of population benchmarks, the 2001 Census of Population and Housing [8] and the population figures used by the Australian Bureau of Statistics for the November 2003 Labour Force Survey (LFS) [9]. Analysis showed little difference between the estimates obtained using these two different benchmarks. The results used in the present analysis are those obtained using the LFS benchmarks, as these refer to the same time period as the survey.

The weight used for the *i*th respondent to the survey was *w*_*i *_= (*N*_*a*_*n*)/*(Nn*_*a*_), where *a *denotes the weighting class and *N *and *n *denote relevant population benchmarks (2001 Census of Population and Housing and LFS) and sample sizes, respectively. Postcodes were used to determine strata for weighting. This allowed determination of the value of the Accessibility Remoteness Index of Australia (ARIA) [10] in which populated localities are given a score from 0 to 12 based on road distance to service towns of different sizes. Scores for regions are derived by averaging scores for localities. Category 1 regions are designated Highly Accessible (ARIA score 0–1.84): relatively unrestricted accessibility to a wide range of goods and services and opportunities for social interaction. Estimates are provided for Category 1 areas and areas in Category 2 or higher.

Because a complex sample design involving stratification and unequal probabilities has been used, the sampling variances of estimates may be different from those that would have been obtained under simple random sampling. Assuming a constant design effect (calculated based on the variation of the weights as 1.47 [11]), Table 2 gives the margins of error for percentages calculated from different sized base populations. Adding and subtracting the indicated margin of error to the estimated result will produce an approximate 95% confidence interval (CI). Approximate 99% CIs can be obtained by multiplying the margin of error by 1.3.

**Table 2:** Margins of error for different percentages and base population sizes and 95% confidence intervals.

Size of Base Population	Percentage				
	
	10	20	30	40	50
3,000	1.3	1.8	2.0	2.2	2.2
2,000	1.6	2.2	2.5	2.7	2.7
1,000	2.3	3.1	3.5	3.8	3.8
500	3.3	4.3	5.0	5.3	5.4
300	4.2	5.6	6.4	6.9	7.0
200	5.1	6.9	7.9	8.4	8.6
100	7.3	9.7	11.1	11.9	12.1

### Questionnaire

Questions from the 1996 BHS were retained as much as possible to allow for the analysis of changes in responses between survey periods. The original questions had been previously tested for validity and reliability (Cohen's kappa statistic, which measures the percentage agreement beyond chance, was *κ *= 0.75 for items relating to respondent behaviour) [2].

The 2003 BHS covered the following themes: knowledge and perceptions about incidence, mortality and risk; knowledge and behaviour regarding early detection, symptoms and diagnosis; mammographic screening; treatment; and accessibility and availability of information and services. Items relating to medicolegal issues were not retained from the 1996 BHS. Ethical approval for the BHS was received from the New South Wales Cancer Council Ethics Committee.

### Statistical analysis

All statistical analyses were undertaken using the appropriate weights. Means and proportions were calculated to give population estimates. Comparisons by age group and area of residence were performed using chi-squared tests. In general, adjustments for complex design and weighting will result in more conservative p-values when compared to unadjusted tests. The level of significance was set at 0.01 and 99% confidence intervals (CIs) were calculated.

## Results

### Participants

A total of 22,740 calls were made (Table 3). Only approximately two out of every five attempts resulted in a response. Of these, approximately two-thirds did not result in an interview. Of the 3,144 of 8,680 (36.2%) respondents who were contacted and consented to being interviewed, 138 (4.4%) had a previous diagnosis of breast cancer and were excluded. The ages of 11 respondents were missing or invalidly coded. These cases were not used in the weighting; the final sample used for calculating weighted estimates was 2,995.

**Table 3:** Summary of call attempts.

Result of Call	Number of Dwelling Attempted	Percentage of Numbers Attempted	Percentage of Eligible Numbers
Interviews	3,006	13	35
Refused the interview	5,044	22	58
Non-English speaking	400	2	5
Respondent not available for study period	230	1	3
No answer after five attempts	2,188	10	
Unusable (disconnected, business, etc.)	4,151	18	
No women aged 30–69	6,757	30	
Other	964	4	
Total	22,740	100	100

### Sociodemographic profile

Respondents had a mean (SD) age of 47.6 (10.2) years, a majority (77.7%) were married or in a *de facto *relationship and about 90% had children (Table 4). Almost three-quarters of all respondents were born in Australia and 1.9% identified themselves as being of Aboriginal or Torres Strait Islander descent.

**Table 4:** Sociodemographic characteristics of respondents.*

Characteristic	Percent (Weighted)
Age, years	
30–39	29.8
40–49	29.5
50–59	24.7
60–69	16.1

Marital status	
Never married	7.1
Married/*de facto*	77.7
Separated but not divorced	3.8
Divorced	7.5
Widowed	3.7
Refused	0.2

Number of children	
None	12.6
1–3	74.5
4 or more	12.9

Country of birth	
Australia	74.4
Overseas	25.6

Indigenous background	
No	98.1
Yes, Aboriginal	1.7
Yes, Torres Strait	0.2

Level of education	
Primary school only	2.0
Less than 3 years of secondary school	7.7
Completed 3–6 years of secondary school	90.3

Qualifications	
None	36.2
Trade	3.8
Certificate or diploma	33.8
Other	25.5
	0.6

Employment	
Full time, paid	33.1
Full time, unpaid	2.4
Part time, paid	30.8
Unemployed, but looking for work	4.1
n Unemployed, but not looking for work	15.1
Retired	14.6

* Results may not sum to 100% due to rounding.

About 98% of respondents had some form of secondary education. About a quarter had a tertiary degree and one-third had a certificate or diploma. About two-thirds of all respondents were in full-time or part-time work.

### General knowledge about mammography

**Figure 1:**
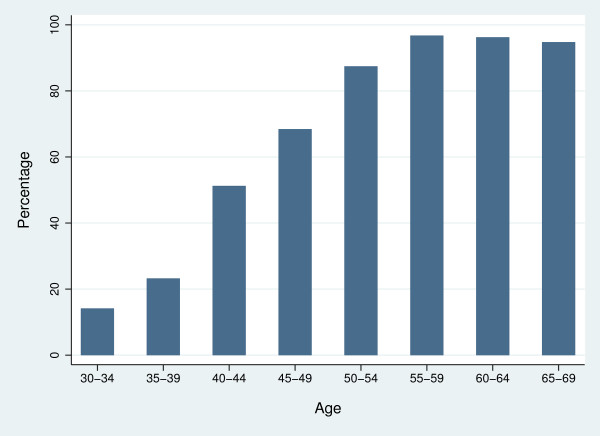
Percentage of women ever having had a mammogram, NBCC Breast Health Survey 2003.

The majority of respondents reported that they had screening mammograms every two years (61.6%). Of the eligible age-group, 42.6% of women aged 40 to 49 and 76% of women aged 50 to 69 reported mammographic screening in the last two years. A similar majority (61.1%, 99% CI 58.3, 63.9) reported to ever having had a mammogram. As expected, this proportion was positively associated with age (Figure 1).

In the context of questions focusing on early detection, women were asked to nominate *"the best way of finding breast cancer"*. Overall, 29.1% (99% CI 26.5, 31.7) identified mammography. Women who reported prior mammogram history were more likely to identify mammography than whose without a prior history (31.3% versus 25.6%; p = 0.005). Older women were more likely to identify mammography (p < 0.001) than younger women. TThere were no differences in responses based on educational level (p = 0.221) or ARIA (p = 0.551).

### Knowledge about BreastScreen Australia

The BreastScreen Australia (BSA) program is the national mammographic screening program providing free biennial screening mammograms for women. Women aged between 50–69 are the "target group", although all women over the age of 40 may participate.

A majority of women (85.9%, 99% CI 83.8, 87.9) had heard of BSA, with recognition being greater for older women: 75.9% (99% CI 71.1, 80.7) in women aged 30–39 versus 92.8% (99% CI 90.4, 95.1) in the target group (p < 0.001). A statistically significantly greater proportion of women living in non-highly accessible areas (ARIA Category 2 or higher) had heard of BSA (93.0%) compared to 84.5% of women living in highly accessible areas (ARIA Category 1, p < 0.001). There were no differences according to educational level (p = 0.724).

A large majority (94.5%, 99% CI 93.1, 95.9) of women believed that BSA attendance is appropriate regardless of the presence or absence of symptoms while 1.0% (99% CI 0.4, 1.6) correctly identified that BSA was a national program for asymptomatic women. There were no significant differences in terms of age (p = 0.172), education (p = 0.208) or ARIA level (p = 0.309).

Women were asked to identify the ages at which BSA *"suggests that women should start *[or stop] *having a mammogram"*. About two of every five participants (41.1%, 99% CI 38.1, 44.1) correctly identified the age of initiation. About three out of every five women (61.4%, 99% CI 58.3, 64.5) were of the opinion that mammographic screening did not have an age ceiling (Table 5). Almost three quarters of women (71.2%, 99% CI 68.5, 74.0) correctly identified that the BSA recommends that screening take place every two years.

**Table 5:** Women's knowledge of the recommended initiation, cessation and frequency of mammographic screening through BreastScreen Australia, NBCC Breast Health Survey 2003.*

Age	Percent (99% CI)
Initiation of screening, 50 years†	41.1 (38.1, 44.1)
	
By decade	
<30	8.1 (6.3, 9.9)
30–39	11.8 (9.8, 13.9)
40–49	36.9 (34.0, 40.0)
50–59	42.5 (39.4, 45.5)
60–69	0.6 (0.2, 1.1)

Cessation of screening, 69 years	0.6 (0.1, 1.1)
	
By decade	
Never stop	61.4 (58.3, 64.5)
<30	0.1 (0.0, 0.4)
30–39	0.1 (0.0, 0.4)
40–49	0.4 (0.0, 0.8)
50–59	1.0 (0.4, 1.7)
60–69	5.4 (3.9, 6.8)
70–79	24.7 (22.0, 27.4)
80+	6.9 (5.3, 8.6)

Frequency of screening	
Biannually	1.4 (0.7, 2.0)
Annually	20.1 (17.7, 22.6)
Biennially†	71.2 (68.5, 74.0)
Less often than biennially	1.9 (1.0, 2.7)
Only when doctor recommends	0.02 (0.0, 0.08)
Other	0.6 (0.0, 1.1)
Can't Say	4.8 (3.5, 6.1)

* Abbreviations: CI, confidence interval; NBCC, National Breasts Cancer Centre.

† Ages and frequency as recommended by the BreastScreen Australia Program.

### Referral and access to the BreastScreen Australia program

Of women aged 50–69, 62.1% (99% CI 57.8, 66.4) reported that their general practitioner recommended that they participate in screening mammography, 84.7% (99% CI 81.4, 88.0) knew that screening mammograms within BSA were free of charge, and 83.6% (99% CI 80.2, 87.0) were "very confident" about where to go or call for an appointment to arrange a screening mammogram.

### Differences between the 1996 and 2003 BHS

There have been substantial gains in women's behaviour and knowledge about mammographic screening over the seven years between the two surveys (Table 6). About 6.1% more respondents in 2003 received a mammogram compared to 1996. In the 2003 BHS, women were more aware that screening occurs every two years (12% point increase). More women in the target age group reported that their general practitioner recommended that they participate in mammographic screening (28% point increase) and more felt "very confident" in arranging a screening appointment (18% point increase). While marginal changes were noted in other areas, 11% more women in the 2003 BHS were able to identify the recommended age at which screening is recommended to commence.

**Table 6:** Relative percentages (99% CI) of selected responses measuring women's knowledge about screening mammography over the course of the 1996 and 2003 NBCC Breast Health Surveys.*

Issue	2003 BHS	1996 BHS	Change from 1996 BHS
Ever had mammogram	61.1 (58.3, 63.9)	55.0 (51.1, 57.9)	6.1

Recognition of BSA†	85.9 (83.9, 87.9)	87.9 (86.0, 89.8)	-2.0

Eligible attendance at BSA			
For asymptomatic women‡	1.0 (0.4, 1.6)	1.3 (0.1, 1.9)	-0.3
Regardless of symptoms	94.5 (93.1, 95.9)	95.0 (93.7, 96.2)	-0.5

Nomination of age of screening initiation			
40–49	36.9 (34.0, 40.0)	26.3 (23.7, 28.9)	10.6
50–59‡	42.5 (39.4, 45.5)	59.4 (56.5, 62.4)	-17.1

Nomination of age of screening cessation			
60–69	5.4 (3.9, 6.8)	9.5 (7.8, 11.2)	-4.1
Never	61.4 (58.3, 64.5)	55.2 (52.2, 58.1)	6.2

Nomination of screening frequency			
Biennially‡	71.2 (68.5, 74.0)	58.7 (55.8, 61.6)	12.5
Annually	20.1 (17.7, 22.6)	26.7 (24.0, 29.4)	-6.6

GP recommended mammographic screening§	62.1 (57.8, 66.4)	34.6 (30.0, 39.3)	27.5

Mammographic screening was free§	84.7 (81.4, 88.0)	84.0 (80.4, 87.6)	0.7

"Very confident" in arranging a screening mammogram	83.6 (80.2, 87.0)	65.8 (61.0, 70.5)	17.8

* Abbreviations: BHS, Breast Health Survey; BSA, BreastScreen Australia; CI, confidence interval; GP, general practitioner.

† In 1996, the national mammographic screening program was known nationally as the National Program for the Early Detection of Breast Cancer.

‡ Recommended by the BreastScreen Australia program.

§ Respondents were aged 50–69.

## Discussion

The NBCC Breast Health Surveys were designed to measure the knowledge, attitudes and behaviour of a nationally-representative sample of women aged between 30 and 69 years. The findings reported in this publication focused on aspects of screening mammography. The results showed areas of improvement while highlighting issues requiring additional development. This, at its core, was the main purpose of the BHS – as an evaluative tool in the continuous improvement of public awareness and health programs in relation to breast health.

About three out of every five age-eligible women interviewed had a mammogram in the past, similar in magnitude to those rates reported elsewhere in the world [12-14], but lower than the target participation set by the BreastScreen Australia Program of 70% [15]. Overall awareness of BSA approached 90%, suggesting that promotion of the national program has been successful. This confirms a general trend toward more widespread recognition of the availability of mammographic screening in Australia [16]. The present results also point to significant differences in awareness by age and area of residence suggesting that future programs, including expanded health promotion campaigns and social marketing activities, should target particular groups.

Equally important were the findings that more than 80% of women knew that the service was free of charge and were "very confident" in arranging a screening mammogram, confirming that community understanding of specific details accompanies its wide promotion and dissemination.

The 28% increase between the 1996 and 2003 BHS in women reporting that their general practitioner had recommended mammographic screening may be due to the success of campaigns directed at general practitioners to encourage screening and early detection in the course of routine care or opportunistically when in consultation with women for other reasons. The coordination of care performed by general practitioners (akin to the North American model of "gatekeeping" primary care physicians [17]) has been shown to increase the uptake of mammographic screening [18-20]. However, the gains in general practitioner participation notwithstanding, more research needs to be undertaken in order to more comprehensively increase the participation rate from the nationwide average (57.1% of the target population in 2001–02 [21]).

The 2003 BHS shows that there is still much confusion about screening as opposed to diagnostic mammography. About 95% of women reported that attendance at BSA was available to women regardless of symptoms. In fact, as for all population screening programs, the BSA program is for asymptomatic women. It is important that this confusion between the management of asymptomatic and symptomatic women be clarified to avoid delays in diagnosing a potentially malignant condition. In addition, previous research has indicated that women who do not appreciate the distinction between diagnostic and screening tests may be more likely to seek compensation for missed diagnoses [22]. Women experiencing symptoms are best managed by undergoing individual assessment and investigations for the cause of the symptoms. The National Breast Cancer Centre recommends the use of a "triple test" of a clinical examination, imaging and/or non-excisional tissue biopsy [23].

Our findings were derived from self-reports. Such data will be affected by the participant's ability to recall events in the past. Moreover, it is well known that survey participants tend to under-report socially unacceptable or unhealthy behaviour and over-report socially desirable ones [24]. We attempted to reduce this effect by assuring confidentiality [25], using indirect question construction techniques [26], and the use of the same questions from the 1996 BHS. This use of the same questions also allowed for comparability across time periods and a retention of consistency of investigation.

While only 35% of eligible numbers resulted in an interview, this figure is not unusual for this type of research. Much has been written about the declining response rates for surveys conducted over the telephone [27-29] For instance, Curtin and colleagues reported that response rates have declined over a 24-year period from 72% in 1979 to 48% in 2003 [30]. This has been due to a number of factors including increased refusals (such as those due to time constraints or the number of survey requests) and declining contact rates (for instance, the current phenomenon of call screening technology through caller ID and answering machines).

Our results are likely to be biased estimates of true population values due to the exclusion of potential respondents who did not have listed telephones or who did not speak English. In addition, respondents who agreed to participate may have more knowledge or experience than those who refused. Moreover, the sampling process selected one woman aged between 30 and 69 from each household and those women living in households with more than one woman in the required age group had a lower chance of selection. This has not been accounted for in the sample weighting. However, an analysis of the 1% sample file of the 2001 Australian Census of Population and Housing showed that only 3.7% of households have more than one woman aged between 30 and 69, so the impact of this feature is likely to be small. Overall, the net effect of such issues is difficult to predict. However, to the extent that these potential considerations show temporal variability, the possibility that the results across both surveys may validly point to true differences in knowledge, attitudes and behaviour cannot be discounted.

## Conclusion

The NBCC Breast Health Surveys provide a valuable picture of the knowledge of Australian women about a range of issues. The present analysis shows significant gains in knowledge and behaviours relating to mammographic screening, while identifying additional areas for targeted improvement, as in the need to better communicate with women about screening and diagnostic services. Further analysis of additional core topic areas (eg., incidence, mortality, risk and treatment) will provide equally noteworthy insight.

## Abbreviations

ARIA, Accessibility Remoteness Index of Australia; BHS, Breast Health Survey; BSA, BreastScreen Australia; CI, confidence interval; GP, general practitioner; LFS, Labour Force Survey; NBCC, National Breast Cancer Centre; SD, standard deviation.

## Competing interests

The author(s) declare that they have no competing interests.

## Authors' contributions

The authors contributed to the study and subsequent manuscript in the following manner: concept and design of the article: EVV, SJ, CN, DI, HZ; data management: SF, DS; data analysis: EVV, SJ, SF, DS, HZ; data interpretation: EVV, SJ, CN, SF, DS, DI, HZ. All authors participated in critically revising drafts for important intellectual content. All authors read and approved the final version of the manuscript.

## Pre-publication history

The pre-publication history for this paper can be accessed here:


